# Immune Dysregulation Is Associated with Neurodevelopment and Neurocognitive Performance in HIV Pediatric Populations—A Scoping Review

**DOI:** 10.3390/v13122543

**Published:** 2021-12-18

**Authors:** Monray E. Williams, Anicia Janse Van Rensburg, Du Toit Loots, Petrus J. W. Naudé, Shayne Mason

**Affiliations:** 1 Human Metabolomics, Faculty of Natural and Agricultural Sciences, North-West University, Potchefstroom 2531, South Africa; niciar@gmail.com (A.J.V.R.); dutoit.loots@nwu.ac.za (D.T.L.); nmr.nwu@gmail.com (S.M.); 2 Department of Psychiatry and Mental Health, University of Cape Town, Cape Town 7701, South Africa; PJW.Naude@uct.ac.za; 3 Neuroscience Institute, University of Cape Town, Cape Town 7701, South Africa

**Keywords:** inflammation, cytokine, pediatric HIV, HIV infection, HIV exposed uninfected (HEU) and HIV-associated neurocognitive disorders, HAND

## Abstract

HIV-1 is known for its complex interaction with the dysregulated immune system and is responsible for the development of neurocognitive deficits and neurodevelopmental delays in pediatric HIV populations. Considering that HIV-1-induced immune dysregulation and its association with neurodevelopmental and neurocognitive impairments in pediatric populations are not well understood, we conducted a scoping review on this topic. The study aimed to systematically review the association of blood and cerebrospinal fluid (CSF) immune markers with neurocognitive deficits and neurodevelopmental delays in pediatric HIV populations. PubMed, Scopus, and Web of Science databases were searched using a search protocol designed specifically for this study. Studies were selected based on a set eligibility criterion. Titles, abstracts, and full texts were assessed by two independent reviewers. Data from the selected studies were extracted and analyzed by two independent reviewers. Seven studies were considered eligible for use in this context, which included four cross-sectional and three longitudinal studies. An average of 130 (±70.61) children living with HIV, 138 (±65.37) children exposed to HIV but uninfected and 90 (±86.66) HIV-negative participants were included across the seven studies. Results indicate that blood and CSF immune markers are associated with neurocognitive development/performance in pediatric HIV populations. Only seven studies met the inclusion criteria, therefore, these limited the number of significant conclusions which could have been made by using such an approach. All considered, the evidence suggests that immune dysregulation, as in the case of adult HIV populations, also has a significant association with neurocognitive performance in pediatric HIV populations.

## 1. Introduction

Human immunodeficiency virus (HIV)-1 is a retrovirus that not only severely suppresses the host immune system, but also causes neurodevelopmental delays and neurocognitive impairment in both adult and pediatric cohorts. In 2018, approximately 1.7 million children (0–14 years) were living with HIV [1]. Most children with HIV-1 live into adulthood, and they present a higher risk of neurodevelopmental [2] and neurocognitive deficits [3,4] in later life, as compared to individuals who contracted HIV as adults. This is due to the extended exposure to HIV-1 and its related effects, including the dysregulated immune system (higher levels of monocyte activating and inflammatory markers). Furthermore, these neurocognitive deficits are present in children even with the initiation of antiretroviral therapy (ART) as early as 5 months old [5,6,7]. Studies indicate that even HIV-negative children born from HIV positive mothers also present with neurodevelopmental delays and neurocognitive impairment [8,9,10], and maternal HIV-1 and treatment status, as well as regimen type, may be contributing factors influencing neurodevelopment in these pediatric populations. The latter findings on the influence of in utero ART exposure on neurocognitive performance are however contradictory, with some studies reporting a significant association [11], and others no association [10,12,13] of neurodevelopmental delays with ART.

Considering the fact that children living with HIV are now surviving into adulthood with ART, it is important to understand the underlying mechanisms of the central nervous system (CNS) pathology, and how this can be used towards improved patient monitoring, neurocognitive impairment prevention, and improved treatment strategies. Several studies have demonstrated that blood and cerebrospinal fluid (CSF) inflammation is associated with neurocognitive deficits seen in adults living with HIV [14,15,16,17,18,19]. It is thought to also likely be the case in children living with HIV for a number of reasons. Firstly, the exposure of the child to the maternal immune system may affect brain development. Studies suggest that maternal immune activation and increased levels of certain inflammatory markers during pregnancy may negatively influence fetal brain development [20,21,22,23]. Secondly, the immune system of the child itself (and due to HIV-1 exposure) may also influence neurocognitive performance [24,25,26]. However, in comparison to HIV-1 studies conducted in adults, much less is known about the association of immune markers with neurocognitive performance in children exposed to and living with HIV. Furthermore, it is not clear which immune markers are most commonly associated with neurocognitive impairment in pediatric HIV populations.

Therefore, in order to gain a better understanding of the association of immune markers with neurocognitive performance in children exposed to and living with HIV, we conducted a scoping review on the topic. The primary aim of the scoping review was to provide commentary on whether peripheral/CSF immune markers are associated with neurocognitive performance in children exposed to HIV and children living with HIV. Secondary aims were to (1) investigate the potential determinants/confounding factors in these associations, (2) determine the extent of the available evidence by reviewing all literature on this topic to date, (3) determine the value of undertaking a full systematic review and meta-analysis, and (4) to summarize and disseminate most relevant research findings to date.

## 2. Methods

### 2.1. Eligibility Criteria

The eligibility criteria for selecting the relevant literature were all studies comprising children living with HIV or children HIV-exposed but uninfected (all medication types included and no cut off for treatment duration) with neuropsychological and medical assessments. Due to the limited number of studies in this field, we included those with perinatally and behaviorally acquired HIV infected, HIV-exposed uninfected (HEU) and HIV-unexposed uninfected (HUU)/HIV-negative populations. The focus/scope of this investigation was children/pediatric cohorts, therefore only study participants of ≤18 years of age were considered (adult studies (>18 years old) were excluded). For comparability of studies, marker measurements needed to be taken from blood/CSF using solid-phase and/or bead array platforms, including enzyme-linked immunosorbent assay (ELISA), chemiluminescence, multiplex, nephelometry or immunoturbidimetric assays for the analysis of cytokines, chemokines, and monocyte-associated immune markers.

Exclusionary criteria were pre-clinical (animal and cell culture models) studies and reviews. Studies investigating samples other than serum, plasma and CSF markers were excluded, as these were considered outside the scope of this study.

### 2.2. Data Sources

PubMed, Scopus, and Web of Science databases were searched for all studies written in English and published up to 21 September 2021. The full search criteria for each database are included in Appendix A. Briefly, the following search conditions were applied to PubMed: (HIV [mh] OR HIV [tw] OR Acquired Immunodeficiency Syndrome [mh] OR “Acquired Immunodeficiency Syndrome” [tw] OR AIDS [tw]) AND (HIV associated neurocognitive disorders [mh] OR HAND [tw] OR neurocognitive [tw] OR cogniti* [tw] OR Executive Function [mh] OR executive [tw] OR Memory [mh] OR memory [tw] OR Attention [mh] OR attention [tw] OR Neuropsychological Tests [mh] OR neurodevelopmental disorders [mh] or neurodevelopment testing [tw] OR Neurocognitive development [tw]) AND (Inflammation [mh] OR inflammation [tw] OR Neurogenic Inflammation [mh] OR neuroinflammation [tw]).

**Figure 1:**
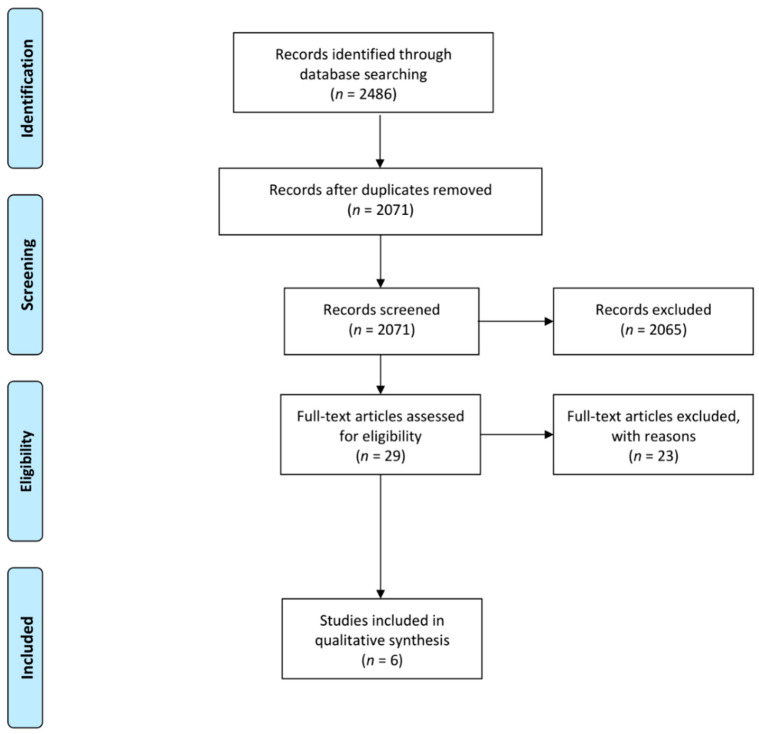
Preferred Reporting Items for Systematic Reviews and Meta-Analyses (PRISMA) flow diagram for results of search strategy.

Furthermore, reference sections were manually searched and the contact authors of the included studies, as well as other experts in this field, were consulted for the inclusion of additional papers. The search strategy and the retrieved articles are shown in Figure 1.

### 2.3. Data Selection

All articles were retrieved and loaded onto a single database using a reference manager (EndNote X9, Clarivate, PA, USA). Two authors, MEW and AJVR, independently identified studies meeting the inclusion criteria. Where there was a discrepancy in article inclusion/exclusion, this was discussed amongst all authors, and a decision was made regarding suitability.

The quality of the included studies was assessed by authors MEW and AJVR and the inter-rater reliability was assessed. The quality criteria were adopted from the Joanna Briggs Institute (JBI) critical appraisal tools, which are used for assessing the trustworthiness, relevance, and results of published papers. For this scoping review, we amended the JBI critical appraisal tools by implementing a Likert scale [27] to provide a quantitative measure of study quality. We only considered the JBI quality questions that may significantly affect findings in the included studies (i.e., inflammatory levels and background information). The aforementioned criteria included the following: (1) potential confounding factors with the appropriate statistical analysis, and (2) background information of the mothers and children living with HIV. These areas were assessed by the following questions:

1. Did the study report on potential confounders and were these controlled for upon statistical analysis, including the controlling of multiple comparisons?
2. Did the study report on the background information of the mother and child, which may have affected inflammatory levels and neurocognitive performance/development in the pediatric population (i.e., maternal substance use, prematurity, ART duration and specific regimens, low birth weight, socioeconomic status and malnutrition)?

Each question was rated as follows: 0 = no, 1 = partly, and 2 = yes. Studies that addressed all the above questions, and had a total rating of 4, were classified as high quality. Studies with a rating between 1 and 3 were considered as intermediate quality, and less than 1 as low quality. A kappa statistic was calculated to measure inter-rater reliability.

### 2.4. Potential Confounders

Several factors may influence the degree of inflammation within the pediatric cohorts investigated. We have therefore investigated the influence of these potential confounders. Firstly, we reviewed the influence of maternal and pediatric viral load and nadir/current CD4^+^ count, in order to determine if the stratification of studies according to these factors determined the association of immune markers with neurocognitive development/performance. Hence, we investigated (1) maternal and (2) pediatric viral load, and (3) nadir and (4) current CD4^+^ count. We stratified the seven studies according to viral load, which was defined as undetectable (viral suppression) versus detectable (non-viral suppression). Next, we stratified according to a mean/median CD4^+^ count of <200 cells/μL or >200 cells/μL. Secondly, we investigated if in utero ART exposure and/or if a specific ART regimen influenced inflammatory profiles. Thirdly, we investigated if maternal inflammation directly, and/or other factors affecting maternal inflammation (i.e., infection during pregnancy), influenced inflammatory profiles in pediatric HIV populations. Fourthly, we investigated the influence of various neurodevelopmental factors (i.e., premature birth, socioeconomic status, and malnutrition) on inflammation. Fifthly, we wanted to determine if any other additional factors could influence the association of the immune markers with neurocognitive development/performance, including gender, the HIV-1 subtype and duration of infection. Lastly, pediatric populations can be grouped into different subpopulations based on age and the developmental stage including neonates (birth to one month), infants (one month to two years), developing children (age 2 to 12 years) and adolescents (12–16 years). It is known that the pediatric immune status differs considerably between these developmental stages [28]. Further, the transplacental transfer of maternal antibodies plays a major role in conferring immune protection in neonates and infants [29,30], and puberty greatly influences the immunological response [31,32]. Therefore, we wanted to determine if immune markers of HIV-1-induced immune dysregulation impacts neurodevelopment when stratifying the studies according to age.

## 3. Results

### 3.1. Study Characteristics

The search strategy yielded a total of 2632 research studies, as indicated in Figure 1. Duplicates (*n* = 433) were removed, resulting in 2199 studies. Thereafter, abstracts and titles were screened and a total of 2175 studies were excluded, which comprised of:

- Review articles/book chapters/conference proceedings (*n* = 731).
- Pre-clinical investigations (*n* = 650).
- Studies not investigating HIV-1 in general (*n* = 193).
- Studies without a neuropsychological evaluation and hence no information on neurocognitive development/performance in children (*n* = 165).
- Participants over the age of 18 (*n* = 141).
- Studies investigating neuroimaging data only (*n* = 59).
- Studies that have not investigated immune markers in general (*n* = 194).
- A study design that required additional blood culturing steps for cytokine measurement (*n* = 1).
- Treatment naïve participants (*n* = 1).
- Studies not reporting statistical analysis for immune marker levels and neurocognitive development/performance (*n* = 1).
- Studies not published in English (*n* = 39), which included: Spanish (*n* = 6), French (*n* = 13), Hebrew (*n* = 2) Japanese (*n* = 3), Chinese (*n* = 2), Polish (*n* = 1), German (*n* = 8), Slovak (*n* = 1), Italian (*n* = 1) and Russian (*n* = 2).

Of the remaining 24 studies, full-text articles were assessed, and an additional 17 were excluded as follows:

- Review (*n* = 1).
- Pre-clinical investigations (*n* = 1).
- Participants over the age of 18 (*n* = 2).
- Studies that have not performed a neuropsychological evaluation to determine neurocognitive development/performance in children (*n* = 6).
- Studies that have not investigated immune markers in general (*n* = 5).
- Studies not reporting statistical analysis for immune marker levels and neurocognitive development/performance (*n* = 2).

Using the selection criteria, 7 studies were eligible for inclusion (Section 2.2), yielding a total sample size of *n* = 783 children living with HIV (mean (SD): 130 (±70.61)), *n* = 207 children exposed to HIV but uninfected (mean (SD): 138 (±65.37)), and *n* = 270 HIV-unexposed/uninfected participants (mean (SD): 90 (±86.66)). Approximately half of the eligible studies (4; 57%) employed a cross-sectional design, while the remainder (3; 43%) employed a longitudinal study design. All participants were on a regimen of ART (exact regimen not stated in all studies) which included the use of first-, second-, and third-line ART. Not all of the studies had reported the minimum ART duration before the relevant assays; however, no restriction was applied for the minimum required duration of ART exposure. Half of the studies reported treatment duration (ranged from birth to 84 months) before analysis. Several immune-related markers were investigated across all 7 studies. To provide practicality (despite this being an oversimplified classification), immune-related markers were clustered into (1) monocyte activation (including neopterin, soluble cluster of differentiation (sCD)14 and sCD163) and (2) inflammation (including C reactive protein (CRP), fractalkine, interferon-γ (IFN)-γ, IFN-α2, IFN-γ-inducible protein-10 (IP-10), interleukin (IL)-1β, IL-2, IL-4, IL-6, IL-10, IL-12, IL-12p40, IL-12p70, macrophage inflammatory protein (MIP)-1α, MIP-1β, monocyte chemoattractant protein (MCP)-1/CCL2, neutrophil gelatinase-associated lipocalin (NGAL), tumor necrosis factor (TNF)-α and thymus and activation regulated chemokine (CCL17).

### 3.2. Neuropsychological Evaluation

Neurocognitive development and performance were evaluated using a range of criteria, including the Bayley Scales of Infant and Toddler Development, Third Edition (*n* = 1), Wechsler Intelligence Scale for Children, Fourth Edition (*n* = 3), Denver Developmental test (*n* = 1), Kaufman Assessment Battery for Children, 2nd edition (KABC-II) (*n* = 1), Tests of Variables of Attention (TOVA) D-prime, Bruininks–Oseretsky Test of Motor Proficiency, 2nd edition (*n* = 1), Behavior Rating Inventory of Executive Function (*n* = 1) and a screening test battery measuring >5 separate composite cognitive domains (*n* = 2).

### 3.3. Quality Assessment of the Included Studies

The kappa was 1.000 and the majority of the included studies were rated as intermediate quality (*n* = 6), with one [33] rated as high quality. As an inclusion criterion, all study participants had to be treated with ART (or initiate treatment in longitudinal studies). Five studies reported on the duration of therapy (ART exposure) before the respective immune marker assays were conducted [24,25,26,33,34]. In addition, all studies reported the exact ART regimen used (Supplementary Table S1). Lastly, only five studies reported on both the duration of treatment before the relevant assays, as well as the exact treatment regimen used [24,25,26,33,34] (Supplementary Table S1). All of the studies have reported and/or controlled for potential covariates within their statistical analysis; however, four studies have controlled for multiple comparisons, which included the Benjamini-Hochberg [26,33], false discovery rate (FDR) [25] and Bonferroni [24]. Only two studies [33,34] provided key background information (e.g., prematurity, low birth weight, ART regiment and treatment duration, maternal health and inflammatory loads) of both mother and child that may have affected the findings. Based on these findings, recommendations are proposed in the latter part of the review.

### 3.4. Immune Markers Associated with Neurodevelopment and Neurocognitive Performance in Pediatric HIV Populations

From the seven selected studies, CRP (5), IL-6 (5), CCL-2 (3), sCD14 (3), and sCD163 (3) were the most reported markers, with the majority of the studies (86%) reporting the association of plasma/CSF immune markers with neurocognitive performance in HIV-pediatric populations (Table 1). Significant associations with neurocognitive performance were reported for several markers, including CRP, CCL2, INF-γ, IL-1β, IL-2, IL-4, IL-6, IL-10, IL-12p70, NGAL, and sCD163 (Table 1). The majority of the immune markers were associated with neurocognitive performance as reported by at least one independent study with IL-6, CRP and sCD163 supported by at least two independent studies. Although we report on a limited number of studies, we considered the markers to be compelling/significant for our purpose when at least 2 independent studies found a consistent direction in the association of the immune marker with neurodevelopment/neurocognitive performance (Table 1). Certain markers were investigated more often and therefore would inherently have more supporting evidence. Therefore, we considered the frequency a marker was investigated when contextualizing the findings. We considered sCD163 to be an important marker for possible future investigation as at least 66% of the studies which included sCD163 in their investigation found it to be associated with neurocognitive impairment in pediatric HIV populations. A descriptive summary of the cohort and main findings of all seven studies are provided in Table 2 and Table 3, respectively.

### 3.5. Potential Confounders

#### 3.5.1. Viral Load

Only one study included data for maternal viral load [33], thus the influence of maternal viral load could not be adequately reported on in the current investigation. Six remaining studies reported viral load in children living with HIV [24,25,26,34,35,36], and the majority of these studies (*n* = 4) included non-virally suppressed participants, 75% of which reported a significant association of immune markers with neurodevelopment/neurocognitive performance. Two studies included virally suppressed participants, and both reported a significant association of immune markers with neurodevelopment/neurocognitive performance [24,34] (Supplementary Table S2).

#### 3.5.2. CD4^+^ Count

Only two studies reported maternal CD4^+^ counts [26,33]; thus, the influence of nadir/current maternal CD4^+^ count could not be investigated. In pediatric populations, only two studies reported the nadir CD4^+^ count [35,36] and two studies reported the current CD4^+^ count [24,35] (all were stratified as having >200 cells/μL). Only one of the two studies reporting nadir CD4^+^ count, and both studies reporting current CD4^+^ count, reported the association of immune markers with neurocognitive performance/development (Supplementary Table S3).

#### 3.5.3. ART, Inflammation, Neurodevelopmental Factors, Gender, HIV-1 Subtype, and Duration of Infection

Only one study has indicated in utero ART exposure [33]. All studies had reported on the exact ART regimen; however, no correlations could be made between the specific regimen, inflammatory profile, or neurocognitive performance. With regards to factors affecting maternal inflammation directly, only one study reported on the maternal inflammatory profile [33] and maternal health, and therefore no conclusions could be made. For various neurodevelopmental factors (i.e., premature birth, socioeconomic status and malnutrition), premature birth was reported in two studies [33,34]; however, no studies have reported data for malnutrition and its influence on neurodevelopment in the current context. Four studies indicated that the participants were from lower socioeconomic status [33,34,35,36]. The remaining studies, however, indicated that participants were from lower-income and/or developing countries, including Kenya [26] and South Africa [24]. Therefore, the majority of the studies were conducted in settings that may be affected by socioeconomic status, and these may have influenced the inflammatory profile [37] and neurocognitive development/performance [38]. Lastly, we wanted to determine if any other additional factors could influence the association of the immune markers with neurocognitive development/performance, including gender and the HIV-1 subtype. Unfortunately, none of the reviewed studies reported data for gender and the HIV-1 subtype in their investigations and a conclusion regarding this could not be made in this study. All HIV-1 pediatric populations were perinatally infected and therefore this review included participants with varying durations of HIV-1 infection. These included participants with HIV-1 infection for <2 years [26,34] or >2 years [24,25,35] at the time of immune maker measurements. Regardless of the duration of HIV-1 infection, all studies reported an association of immune markers with neurocognitive performance/development in pediatric HIV populations.

#### 3.5.4. Age

Three studies included participants with infants [26,33,34] and two with developing children [24,35] and adolescents respectively [25,36]. Regardless of the age stratification, all age subpopulations reported an association of immune markers with neurocognitive performance/development, which suggests that the dysregulated immune response may contribute to neurocognitive outcomes as early as infancy into adolescence. This study could not link particular immune markers to specific age groups because similar immune markers were not investigated across the limited seven studies.

## 4. Discussion

Despite the limited number of studies in the field of pediatric HIV research, the main finding from this review suggests that aberrant immune dysregulation (due to higher levels of monocyte activating and pro-inflammatory markers) is associated with neurocognitive performance in children living with HIV, and children HIV-exposed but uninfected. It is of interest to note that 71% of the selected studies were published in the last 2 years, and all studies within the last 10 years. Based on the limited evidence and information available, at this stage, it was not possible to conduct a full systematic review and meta-analysis. However, there are several important findings made in this review.

Firstly, the markers related to monocyte activation and inflammation were associated with neurocognitive performance in children living with HIV and in children HIV-exposed but uninfected across several studies. These findings indicate that as in adults living with HIV, inflammation is associated with neurocognitive impairment in children living with HIV. Furthermore, even in the absence of HIV-1 infection, maternal HIV-1 infection may influence the dysregulation of inflammation in pediatric populations, and this in turn impairs neurodevelopment [33]. Considering the above, these findings support the role of the dysregulated peripheral/CNS immune system (higher levels of monocyte activating and pro-inflammatory markers due to HIV-1 exposure) in the development of neurocognitive impairments/delays in pediatric HIV populations. Interestingly, CRP, IL-6 and sCD163 were the only markers whose association with neurocognitive impairment was supported by at least two of the seven independent studies, and for this reason, we consider these markers to be compelling for future investigations. We also took into consideration the frequency a marker has been investigated across all studies. The findings indicate that sCD163 is an important marker for future investigation as at least 66% of the studies which included sCD163 in their investigation found it to be associated with neurocognitive impairment in pediatric HIV populations. sCD163 is an immune indicator for monocyte activation [39] and therefore highlights the relevance of an activated immune system in pediatric HIV populations. In recent systematic reviews conducted by our group, peripheral [40] and CSF sCD163 levels [41] were consistently associated with neurocognitive impairment in adults living with HIV. HIV-1 invades the CNS through infected monocytes and the activity of the HIV-1 within the CNS significantly dysregulates the immune response [42]. The dysregulated immune activation and low-grade inflammation are considered to be key contributors to the development of cognitive deficits in adults [43] and children [44,45] within the modern ART era. This review supports the findings from previous studies in adults [18,39,46,47], indicating that even at younger ages, persistent monocyte activation (sCD163) and inflammation (CRP and IL-6) are potentially key pathways in the development of neurocognitive deficits and may be targeted in developing alternative treatment strategies. The neuropathology of HIV-1 is generally the same for adults and children [42,48,49]; however, the effects of HIV-1 in the CNS differs when comparing these groups. It has been suggested that children have a more (1) florid inflammation, (2) higher frequency of multinucleated giant cells in the cerebral cortex and (3) more basophilic mineralization [50]. Adults have more perivascular brown pigments and more obvious white matter changes [50]. The frequency of physical brain damage for children vertically infected is greater compared to adults living with HIV [51]. Further, substantial brain development in the first few years of life places children living with HIV at greater risk of developing neurocognitive impairment compared to adults [52,53]. In particular, it was shown that language functions are more impaired in children living with HIV compared to adults living with HIV [54], which supports the premise of the phenomenon of increased virulence of viral infections in the immature CNS [50,55]. Studies that directly compare inflammatory profiles between adults and children living with HIV remain limited.

Furthermore, we have particularly focused on blood and CSF immune markers due to the (1) ease and limited invasiveness of collecting these samples compared to other sample types (i.e., postmortem brain tissue), and (2) the fact that these samples may provide insight into the biochemical milieu of the immune system under certain disease states.

Secondly, we report the potential determinants that may affect the associations between the immune markers and neurodevelopmental outcomes. From the limited evidence available, the association of immune markers with neurocognitive development/performance was persistent regardless of CD4^+^ count and viral load. This potentially represents an unchecked monocyte activation and inflammation, and this might be an initiator for ongoing neuroinflammation and cognitive impairment. ART may be ineffective in reducing the viral load matching pre-infection levels [56] and this further suggests that ART alone may not be sufficient to resolve HIV-1-induced neuroinflammation and the subsequent development of HIV-associated neurocognitive impairment in the modern ART era. Considering that children living with HIV are expected to live for a longer period with HIV-1 compared to adults contracting it in later life, this may suggest that these children may also experience an extended exposure to the dysregulated immune system (higher levels of monocyte activation and potentially pro-inflammatory environments). This may result in a greater risk of developing neurocognitive deficits and increased neurocognitive severity in later life. Longitudinal studies are required for this purpose.

Thirdly, we report that studies in this field are extremely limited. This emphasizes the growing interest in this topic and the need for further investigations, since an understanding of the role of the HIV-1 dysregulated immune system in children over time may serve to elucidate the mechanisms related to the development of HIV-associated neurocognitive disorders.

Lastly, this review provides an overview of studies to date, reporting the association of immune markers with neurocognitive performance in pediatric HIV populations, and makes suggestions for the planning and execution of similar studies undertaken on this topic in the future.

## 5. Recommendations

Based on the findings of the quality assessment, several suggestions could be made to improve the quality of future studies in this area. Firstly, studies should include the (1) duration of HIV-1 infection before participants started ART and (2) duration of treatment before the assessment of immune marker levels where ART was not implemented at birth, since an early initiation of ART was associated with many beneficial effects on multiple markers of immune activation, inflammation and viral persistence [57,58,59]. Furthermore, in utero ART exposure, duration, and specific ART regimens may also influence inflammatory levels reported in the HIV pediatric cohorts; therefore, these need to be reported as they may help contextualize the association of markers with HIV-associated neurocognitive impairment. Secondly, neuroinflammatory marker levels and the prevalence of neurocognitive delays may be confounded by several factors, as highlighted throughout the review. Therefore, studies need to report these potential confounders as well as control for them upon statistical analysis. Studies should also control for multiple comparisons. Thirdly, future studies should investigate all relevant maternal related factors (i.e., maternal health, inflammatory loads, ART duration and regimens) in order to contextualize the inflammatory profiles in pediatric HIV populations. Fourthly, in studies investigating inflammation, cognizance needs to be taken of potential neurodevelopmental factors (i.e., premature birth, socioeconomic status, and malnutrition) which may influence inflammatory levels. Finally, studies should develop a uniform pipeline for investigating inflammation and neurodevelopment/neurocognitive performance, in order to limit the potential confounding factors when interpreting findings. This will allow for improved comparability of studies and create a clearer picture of the association of immune markers with neurodevelopment/neurocognitive performance in pediatric HIV populations.

## 6. Limitations

We felt it necessary to briefly mention possible limitations to this investigation. Firstly, seven studies met the inclusion criteria; therefore, this limited the number of significant conclusions which could have been made if more studies were available on this topic. As an additional aim, we wanted to investigate the possible influence of several factors, including maternal and neurodevelopmental factors, age, gender, HIV-1 subtype, viral suppression, duration of infection and CD4^+^ count on neurodevelopment in pediatric HIV populations; however, such data were not sufficiently reported, and no conclusions could be made. Secondly, the majority of the included studies have not declared HIV-1 subtype status. Considering the HIV-1 subtype may affect the severity of (1) neurocognitive impairment in pediatrics [60] and adults [61] and (2) inflammation [62], this information becomes important in contextualizing the reported associations.

Another important factor to consider is that despite the well-defined inclusionary criteria used in the selection of literature in this review, heterogeneity was still evident. This heterogeneity can be explained by two factors. Firstly, the different studies employed various measures of neurodevelopment and neurocognitive performance and the same neurodevelopmental assessment used may not necessarily be universal and effective across all geographical regions [63]. Secondly, there is also a significant variation in cytokine levels between techniques of immune marker measurements [64,65,66], and these variations may lead to inconsistent associations observed in the reported studies. Lastly, although this review reported that the majority of the studies showed an association of the analyzed immune markers with neurodevelopment/performance, these studies report findings of selected immune markers (based on evidence of its involvement in the neuropathophysiology of HIV-associated neurocognitive impairment), and therefore the findings may present a selection bias. Other exploratory investigations may have reported findings for markers that are not presented here, which may also be of value.

## 7. Conclusions

Here we report from the available evidence that immune dysregulation is associated with neurodevelopment and neurocognitive performance in HIV pediatric populations. Several immune markers were associated with neurodevelopment and neurocognitive performance, with findings for CRP, IL-6, and sCD163 supported by at least two of the seven selected independent studies. Furthermore, sCD163 was considered an important marker for future investigation since the majority of the studies that selected sCD163 in its investigation found it to be associated with neurocognitive impairment in pediatric HIV populations. The association of immune markers with neurocognitive impairment were reported regardless of CD4^+^ count and viral load. This investigation also illustrated the limited amount of data available on the topic, emphasizing the need for further investigation. Based on our findings, we suggest the latter be conducted using uniform investigative approaches, as these may help develop a consensus in the immune markers analyzed and their association with neurocognitive development and impairment in HIV pediatric populations.

## Figures and Tables

**Table 1:** Immune markers associated neurodevelopment delays and neurocognitive impairment in pediatric HIV populations.

Markers	Levels	*p*-Value (*p*)/Effect Size	Domain Affected	Reference
Serum-C Reactive Protein (CRP)	Higher	*p* < 0.01, r = −0.33	general intelligence	[24]
	Higher	*p* = 0.03, r = −0.24	visual spatial ability	
	Higher	*p* = 0.04, r = −0.22	executive function	
Plasma-C Reactive Protein (CRP)	Higher (part of a clustered group of investigated markers)	Kaufman Assessment Battery for Children (KABC)-II: week 0: (Regression Coefficient (RC): −2.19 (−3.67, −0.71)) Week 48 (CI: −3.04 (−4.73, −1.34)) Test of Variables of Attention (TOVA) D-prime: Week 0 (RC: −0.22 (−0.36, −0.07)) Week 48: (RC: −0.18 (−0.34, −0.03))	cognition and attention/impulsivity	[34] *
Cerebrospinal fluid (CSF)–Chemokine (C-C motif) ligand 2(CCL2)	Lower	*p* = 0.004	processing speed	[25]
Serum-Interferon-γ (IFN-γ)	Higher	*p* = 0.011, r = −0.339	motor development	[33] *
Serum-Interleukin-1β (IL-1β)	Higher	*p* < 0.001, r = −0.491	motor development	[33] *
Serum-IL-2	Higher	*p* = 0.022, r = −0.308	motor development	[33] *
Serum-IL-4	Higher	*p* = 0.002, r = −0.418	motor development	[33] *
Serum-IL-6	Higher	*p* = 0.004, r = −0.383	motor development	[33] *
	Higher	HIV+ and HIV exposed uninfected (HEU): *p* = 0.014, estimate = −0.19 HIV+: *p* = 0.032, estimate = −0.21	processing speed	[35]
Serum-IL-10	Higher	*p* = 0.001, r = −0.451	motor development	[33] *
Serum-IL-12p70	Higher	*p* = 0.004, r = −0.379	motor development	[33] *
Serum-Neutrophil gelatinase-associated lipocalin (NGAL)	Higher	*p* = 0.004, r = −0.383	motor development	[33] *
Plasma-Soluble cluster of differentiation (sCD163)	Higher	*p* = 0.07, r = −0.42	cognition	[26] *
	Higher	*p* = 0.08, r = −0.52	short-term memory	
	Higher	*p* = 0.05, r = −0.39	non-verbal test performance	
	Higher (part of a clustered group of investigated markers)	KABC-II: week 0: (RC: −2.19 (−3.67, −0.71)) Week 48 (CI: −3.04 (−4.73, −1.34)) TOVA D-prime: Week 0 (RC: −0.22 (−0.36, −0.07)) Week 48: (RC: −0.18 (−0.34, −0.03))	cognition and attention/impulsivity	[34] *
Plasma Macrophage inflammatory protein (MIP)-1β	Lower (part of a clustered group of investigated markers)	TOVA D-prime: Week 48 (RC: 0.17 (0.02, 0.33)) Between week 0 and 48 (RC: 0.20 (0.08, 0.33)).	attention/impulsivity	[34] *

* Longitudinal study design. Abbreviations: CCL2 (chemokine (C-C motif) ligand 2), CRP (C-reactive protein), HIV-1 exposed uninfected (HEU), interferon (IFN), interleukin (IL), Kaufman Assessment Battery for Children (KABC), macrophage inflammatory protein (MIP), neutrophil gelatinase-associated lipocalin (NGAL), regression coefficients (RC), soluble cluster of differentiation (sCD) and Test of Variables of Attention (TOVA).

**Table 2:** Summary of cohort information of studies reporting the association of immune markers with neurodevelopment and neurocognitive performance in pediatric HIV populations.

Reference	Cohort	Sample Type	ART	Assessment of Neurodevelopment/Performance	Markers Investigated	Method	Covariates
[26] *	Perinatal HIV (PHIV)+: *n* = 67 (31 male) Age: 3.8 (3.1–4.0) months Baseline Plasma VL 3,981,072 (1,000,000–10,000,000) copies/mL CD4 count: N/A	Blood	Antiretroviral therapy (ART) initiation at study entry. Ritonavir-boosted lopinavir (if previously exposed to nevirapine) or nevirapine combined with lamivudine and zidovudine. Infants enrolling later received abacavir instead of zidovudine.	Milestones were selected and adapted based on items in the Denver Developmental Screening Test. NPT testing battery included: (1) Kaufman Assessment Battery for Children 2nd edition (2) Behaviour Rating Inventory of Executive Functioning (3) Visual Test of Variables of Attention (4) Bruininks–Oseretsky Test of Motor Proficiency 2nd edition.	sCD163, sCD14 and Neopterin	Enzyme linked immunosorbent assay (ELISA)	6-month plasma HIV RNA level, CD4%, and weight-for-age.
[25]	PHIV+: *n* = 34 (16 male) HIV-: *n* = 37 (18 male) Age: PHIV: 13.6 (11.5–15.9) years HIV-: 12.1 (11.5–15.7) years CD4 count: N/A Plasma and CSF VL: 83% < 150 copies/mL	Plasma and CSF	cART (Protease inhibitors, Abacavir)	Wechsler Intelligence Scales for Children (age ≤ 15 years) Wechsler Adult Intelligence Scales (age ≥ 16 years)	Plasma: CCL2, INF-ƴ, IP-10, CRP, sCD14, sCD163 CSF: CCL17, IL-12p40, MIP-1-alpha, IL-6, CCL2 and sCD163	Meso Scale Discovery electrochemiluminescence-based immunoassay, ELISA	Age, sex, ethnicity, and socio-economic status.
[24]	PHIV+: *n* = 168 (81 male) HIV-: *n* = 43 (18 male) Age: PHIV: 10.8 (±0.89) months HIV-: 10.7 (±0.99) months CD4 count: Current: 980 (±503) cells/µL Plasma VL: 0 (±40) copies/mL	Blood	ART > 6 months: (First/second/third line)	Child Behaviour Checklist, functional competence and a battery of tests measuring 10 separate composite cognitive domains: general intellectual, functioning, attention, working memory, visual memory, verbal memory, language, visual-spatial ability, motor coordination, processing speed, and executive function.	CRP	Immunoturbidimetric assay	Age
[36]	PHIV+: *n* = 89 (39 male) Age: 12.3 (10.5–14.12) years VL: 251,189 (100,000–630,957) copies/mL Nadir CD4 count: 366 (184–595) cells/µL	Blood	HAART: 91%	Wechsler Intelligence Scale for Children-Fourth Edition (WISC-IV)	CCL2, IL-6, and CRP	ELISA and Nephelometry	Demographics, disease severity, and receipt of HAART
[35]	PHIV+: *n* = 212 (88 male) HEU: *n* = 130 (70 male) Age: 11.4 (±2.60) years CD4 count: Nadir: 386 (220–586) cells/µL Current: 713 (495–920) cells/µL Viral load (VL): 398 (50–1995) copies/mL	Serum	HAART- (protease inhibitors and non-nucleoside reverse transcriptase inhibitors)	WISC-IV	CRP, IL-6 and CCL2	ELISA and Nephelometry	Multiple
[34] *	PHIV+: *n* = 213 (92 male) Age: 1.2 (0.5–2.6) years CD4 count: N/A Viral load: 100% < 400 copies/mL	Plasma	ART (nevirapine, lopinavir/ ritonavir)	NPT testing battery included: (1) Kaufman Assessment Battery for Children, 2nd edition (KABC-II) Mental Processing Index (2) Tests of Variables of Attention (TOVA) D-prime (3) Bruininks–Oseretsky Test of Motor Proficiency, 2nd edition (4) Behavior Rating Inventory of Executive Function, Parent Form, Global Executive Composite	CRP, IL-10, CX3CL1, MCP-1, MIP-1β, IFN-γ, IFN-α2, IL-1β, IL-6, IP-10, TNFα sCD14, sCD163	ELISA	Age and sex, study site, sex, age at study entry, age at ART initiation, ART regimen at the time of biomarker specimen collection participant regimens, specimen collection and consensus factor scores at week 0.
[33] *	Infants (6–10 weeks): HEU *n* = 63 (26 male) HUU: *n* = 159 (70 male) HEU children (24–28 months): *n* = 77 (30 male) HUU children: *n* = 190 (77 male) CD4 count: N/A	Serum	Prophylaxis (nevirapine alone or combined with zidovudine) from birth	Bayley Scales of Infant and Toddler Development, third edition (24–28 months)	IFN-γ, IL-1β, IL-2, IL-4, IL-5, IL-6, IL-7, IL-8, IL-10, IL-12p70, IL-13, TNF-α and NGAL	Multiplex bead array and ELISA	Maternal sociodemographic and lifestyle factors, infant health, and maternal HIV disease parameters.

* Longitudinal study design. Abbreviations: ART (antiretroviral therapy), cART (combination antiretroviral therapy), CCL17 (thymus and activation-regulated chemokine), CCL2 (chemokine (C-C motif) ligand 2), CRP ( C-reactive protein), CSF (cerebrospinal fluid), HAART (highly active antiretroviral therapy), HEU (HIV exposed uninfected), HUU (HIV unexposed uninfected), IFN-γ (interferon gamma), IL-10 (interleukin-10), IL-12p40 (interleukin-12p40), IL-12p70 (interleukin-12p70), IL-13 (interleukin-13), IL-1β (interleukin-1β), IL-2 (interleukin-2), IL-4 (interleukin-4), IL-5 (interleukin-5), IL-6 (interleukin-6), IL7 (interleukin-7), IL-8 (interleukin-8), NGAL (neutrophil gelatinase-associated lipocalin), sCD14 (soluble cluster of differentiation 14), sCD163 (soluble cluster of differentiation 163), TNF-α (tumor necrosis factor-α) and viral load (VL). Multiple: Age, sex, race, ethnicity, primary language, caregiver education, household income, relationship of participant and caregiver, hyperlipidemia status, BMI z-score, serum lipids, body composition measures, fasting glucose and insulin, log homeostasis model assessment of insulin resistance, receipt of HAART, protease inhibitors or non-nucleoside reverse transcriptase inhibitors, CDC HIV disease class, current CD4+ cell count, current and nadir CD4+ percentage (CD4%), log viral load, and log peak viral load.

**Table 3:** Main findings of studies reporting the association of immune markers with neurodevelopment and neurocognitive performance in pediatric HIV populations.

Reference	Main Findings
[26] *	No associations were found between plasma concentrations of neopterin or sCD14 before or after ART initiation and neurodevelopmental outcomes (both *p* > 0.05). Infants with high entry plasma sCD163 concentration had an earlier age at attainment of sitting supported (*p* = 0.006) and walking supported (*p* = 0.02). Infants with high sCD163 at 6 months after initiating ART were older at the achievement of milestones compared to those with low sCD163 (speech: *p* = 0.02, threw toys: *p* = 0.04). Before ART, monocyte activation may reflect transient neuroprotective mechanisms in infants. After ART and viral suppression, monocyte activation may predict worse short- and long-term neurodevelopment outcomes. 27 of the 67 children were followed for 5.8–8.2 years. Nine of 27 children had elevated sCD163 at 6 months after initiation of ART and had worse global cognitive ability (*p* = 0.07, r = −0.42), short term memory (*p* = 0.08, r = −0.52), nonverbal test performance (*p* = 0.05, r = −0.39).
[25]	Children living with HIV had higher plasma levels of CRP (*p* = 0.035), INF-ƴ (*p* = 0.021), IP-10 (*p* = 0.035), and CCL2 (*p* = 0.004) compared to HIV negative controls. CSF CCL2 positively associated with processing speed (*p* = 0.004). The association between higher CSF sCD163 and NFH could indicate a relationship between monocyte activation and axonal damage. Overall, markers of immune activation and inflammation seem to be associated more frequently and strongly with CNS injury in cART-treated children than conventional HIV-1 VL measurements or (nadir) CD4+ T-cell counts.
[24]	There were significant differences between the HIV+ and control groups in domains: general intellectual functioning (*p* = 0.001, T = 3.46), executive functioning (*p* ≤ 0.001, T = 3.82), working memory (*p* ≤ 0.001, T = 4.22), verbal memory (*p* = 0.002, T = 3.25) visual memory (*p* = 0.002, T = 3.09), language (*p* = 0.048, T = 2.00), processing speed (*p* ≤ 0.001, T = 4.57). No significant differences between groups in motor coordination (*p* = 0.776, T = −0.29) attention (*p* = 0.059, T = 1.90) and visual spatial ability (*p* = 0.052, T = 1.96). HIV+ group had significantly lower scores on the Child Behaviour Checklist functional competence 3 Cognitive domains were significantly negatively associated with CRP: general intelligence (*p* ≤ 0.01, r = −0.33), visual spatial acuity (*p* = 0.03, r = −0.24), and executive function (*p* = 0.04, r = −0.22). The controls and HIV+ without a neurocognitive disorder had no significant correlations between CRP and cognitive functioning domains (*p* ≥ 0.05 for all domains).
[36]	None of the markers was associated with the Full-Scale IQ test (all *p* > 0.05)
[35]	Individually, none of the nine candidate biomarkers showed significant relationships with FSIQ in linear regression models (all *p* > 0.05) Both HIV+ and HEU mean WISC-IV scores were in the low average range but did not differ significantly by HIV status (*p* > 0.40 for all biomarkers). Using factor analysis, a significant negative association of a group of markers (CRP, IL-6, and fibrinogen) were associated with WISC-IV processing speed score (estimate = −0.21, *p* = 0.032). This finding persisted when restricted to PHIV+ participants after adjusting for peak viral load, nadir CD4%, non-English exposure, BMI z-score, caregiver education, and household income.
[34] *	Higher Factor B (sCD163, sICAM-1, sVCAM-1, CRP) scores, at week 0, were associated with lower (poorer) KABC-II scores at weeks 0 (regression coefficient (RC): −2.19 (−3.67, −0.71) and 48 (CI: −3.04 (−4.73, −1.34) and poorer TOVA D-prime scores at weeks 0 (RC: −0.22 (−0.36, −0.07) and 48 (RC: −0.18 (−0.34, −0.03). Higher Factor D (MIP-1β, VEGF-A); scores, at week 0, were associated with higher (better) TOVA D-prime scores at week 48 (RC: 0.17 (0.02, 0.33) and a greater increase in TOVA D-prime scores between weeks 0 and 48 (RC: 0.20 (0.08, 0.33). Higher Factor E (sCD14, CRP) scores at week 0, associated with increased KABC-II scores between weeks 0 and 48 (RC: −1.17 (−2.18, −0.16). Higher Factor D (MIP-1β, VEGF-A) scores assessed at the start of controlled viremia was associated with higher TOVA D-prime values at Week 0. At week 48, higher Factor B (sCD163, sICAM-1, sVCAM-1, CRP) scores were associated with poorer KABCII scores (RC: −3.04 (−4.73, −1.34).
[33] *	No inflammatory markers in mothers with HIV were significantly associated with neurodevelopmental measures in HEU children (all *p* > 0.05). HIV-1 infection was associated with lower serum levels of IFN-γ (*p* = 0.006) and IL-1β (*p* = 0.006) in HEU children at 6–10 weeks. HIV-1 infection was associated with lower serum levels of IFN-γ (*p* = 0.006), IL-1β (*p* < 0.001), IL-2 (*p* = 0.004) and IL-4 (*p* = 0.013) in HEU children at 24–28 months. In HEU children at 6–10 weeks, higher levels of IFN-γ (*p* = 0.011, r = −0.339), IL−10 (*p* = 0.001, r = −0.451), IL-12p70 (*p* = 0.004, r = −0.379), IL-1β (*p* < 0.001, r = −0.491), IL-2 (*p* = 0.022, r = −0.308), IL-4 (*p* = 0.002, r = −0.418), IL-6 (*p* = 0.004, r = −0.383) and NGAL (*p* = 0.004, r = −0.383) were associated with poorer motor development after controlling multiple comparisons and covariates on all models.

* Longitudinal study design. Abbreviations: ART (antiretroviral therapy), body mass index (BMI), CCL2, (chemokine (C-C motif) ligand 2), confidence interval (CI), CNS (central nervous system), CRP (C-reactive protein), full fcale intelligence quotient (FSIQ), HIV-1 exposed uninfected (HEU), interferon (IFN), interferon γ-induced protein (IP-10), interleukin (IL), Kaufman Assessment Battery for Children (KABC), macrophage inflammatory protein (MIP), neurofilament heavy-chain (NFH), neutrophil gelatinase-associated lipocalin (NGAL), regression coefficients (RC), soluble cluster of differentiation (sCD), soluble intercellular adhesion molecule-1 (sICAM-1), soluble vascular cell adhesion molecule-1 (sVCAM-1) Test of Variables of Attention (TOVA), viral load (VL) and Wechsler Intelligence Scale for Children (WISC).

## Supplementary Materials

The following are available online at https://www.mdpi.com/article/10.3390/v13122543/s1, Table S1: (1) Quality assessment of studies by M.E.W., (2) Quality assessment of studies by A.J.V.R.; Table S2: Studies reporting a relationship of immune markers and HIV-associated neurocognitive performance/development in pediatric populations when stratified according to viral suppression; Table S3: Studies reporting a relationship with HIV-associated neurocognitive performance when stratified according to nadir/current CD4 count.

## Appendix A

Full search criteria

Pubmed (*n* = 1447) 21/09/2021

(HIV [mh] OR HIV [tw] OR Acquired Immunodeficiency Syndrome [mh] OR “Acquired Immunodeficiency Syndrome” [tw] OR AIDS [tw]) AND (HIV associated neurocognitive disorders [mh] OR HAND [tw] OR neurocognitive [tw] OR cogniti* [tw] OR Executive Function [mh] OR executive [tw] OR Memory [mh] OR memory [tw] OR Attention [mh] OR attention [tw] OR Neuropsychological Tests [mh] OR neurodevelopmental disorders [mh] or neurodevelopment testing [tw] OR Neurocognitive development [tw]) AND (Inflammation [mh] OR inflammation [tw] OR Neurogenic Inflammation [mh] OR neuroinflammation [tw]).

Scopus (*n* = 514) 21/09/2021

(HIV OR acquired immunodeficiency syndrome OR “acquired immunodeficiency syndrome” OR aids) AND (hiv associated neurocognitive disorders OR hand OR neurocognitive OR cogniti* OR executive function OR executive OR memory OR attention OR neuropsychological tests OR neurodevelopmental disorders OR neurodevelopment testing OR Neurocognitive development) AND (cytokines OR cytokin* OR chemokines OR inflammation OR neurogenic inflammation OR neuroinflammation OR tnf OR interleukins) AND (microglia OR monocytes OR monocyte* OR scd163 OR scd14 OR scd40 OR neopterin OR interferons).

Web of Science (*n* = 671) 21/09/2021

TS = (HIV OR Acquired Immunodeficiency Syndrome OR” Acquired Immunodeficiency Syndrome” OR AIDS) AND TS = (HIV associated neurocognitive disorders OR HAND OR neurocognitive OR cogniti* OR Executive Function OR executive OR Memory OR memory OR Attention OR attention OR Neuropsychological Tests neurodevelopmental disorders OR neurodevelopment testing OR Neurocognitive development) AND TS = (Cytokines OR cytokin* OR Chemokines OR chemokine OR Inflammation OR inflammation OR Neurogenic Inflammation OR neuroinflammation OR TNF OR Interleukins OR interleukins) AND TS = (Microglia OR microglia OR Monocytes OR monocyte* OR sCD163 OR sCD14 OR sCD40 OR neopterin OR interferons).

Total: 2632

## References

[1] UNAIDS (2020). UNAIDS Global Hiv Statistics 2020. Ending AIDS Epidemic.

[2] Chase C., Ware J., Hittelman J., Blasini I., Smith R., Llorente A., Anisfeld E., Diaz C., Fowler M.G., Moye J. (2000). Early cognitive and motor development among infants born to women infected with human immunodeficiency virus. Women and Infants Transmission Study Group. Pediatrics.

[3] Phillips N., Amos T., Kuo C., Hoare J., Ipser J., Thomas K.G.F., Stein D.J. (2016). HIV-associated cognitive impairment in perinatally infected children: A meta-analysis. Pediatrics.

[4] Laughton B., Cornell M., Boivin M., Van Rie A. (2013). Neurodevelopment in perinatally HIV-infected children: A concern for adolescence. J. Int. AIDS Soc..

[5] Koekkoek S., Eggermont L., De Sonneville L., Jupimai T., Wicharuk S., Apateerapong W., Chuenyam T., Lange J., Wit F., Pancharoen C. (2006). Effects of highly active antiretroviral therapy (HAART) on psychomotor performance in children with HIV disease. J. Neurol..

[6] Puthanakit T., Ananworanich J., Vonthanak S., Kosalaraksa P., Hansudewechakul R., Van Der Lugt J., Kerr S.J., Kanjanavanit S., Ngampiyaskul C., Wongsawat J. (2013). Cognitive function and neurodevelopmental outcomes in HIV-infected children older than 1 year of age randomized to early versus deferred antiretroviral therapy: The PREDICT neurodevelopmental study. Pediatr. Infect. Dis. J..

[7] Whitehead N., Potterton J., Coovadia A. (2014). The neurodevelopment of HIV-infected infants on HAART compared to HIV-exposed but uninfected infants. AIDS Care.

[8] Le Roux S.M., Donald K.A., Brittain K., Phillips T.K., Zerbe A., Nguyen K.K., Strandvik A., Kroon M., Abrams E.J., Myer L. (2018). Neurodevelopment of breastfed HIV-exposed uninfected and HIV-unexposed children in South Africa. AIDS.

[9] McHenry M.S., McAteer C.I., Oyungu E., McDonald B.C., Bosma C.B., Mpofu P.B., Deathe A.R., Vreeman R.C. (2018). Neurodevelopment in Young Children Born to HIV-Infected Mothers: A Meta-analysis. Pediatrics.

[10] Wedderburn C.J., Yeung S., Rehman A.M., Stadler J.A.M., Nhapi R.T., Barnett W., Myer L., Gibb D.M., Zar H.J., Stein D.J. (2019). Neurodevelopment of HIV-exposed uninfected children in South Africa: Outcomes from an observational birth cohort study. Lancet Child Adolesc. Health.

[11] Rice M.L., Russell J.S., Frederick T., Purswani M., Williams P.L., Siberry G.K., Redmond S.M., Hoffman H.J., Yao T.J. (2018). Risk for Speech and Language Impairments in Preschool Age HIV-exposed Uninfected Children with In Utero Combination Antiretroviral Exposure. Pediatr. Infect. Dis. J..

[12] Chaudhury S., Mayondi G.K., Williams P.L., Leidner J., Shapiro R., Diseko M., Ajibola G., Holding P., Tepper V., Makhema J. (2018). In-utero exposure to antiretrovirals and neurodevelopment among HIV-exposed-uninfected children in Botswana. AIDS.

[13] Boivin M.J., Maliwichi-Senganimalunje L., Ogwang L.W., Kawalazira R., Sikorskii A., Familiar-Lopez I., Kuteesa A., Nyakato M., Mutebe A., Namukooli J.L. (2019). Neurodevelopmental effects of ante-partum and post-partum antiretroviral exposure in HIV-exposed and uninfected children versus HIV-unexposed and uninfected children in Uganda and Malawi: A prospective cohort study. Lancet HIV.

[14] Gougeon M.L., Poirier-Beaudouin B., Durant J., Lebrun-Frenay C., Saïdi H., Seffer V., Ticchioni M., Chanalet S., Carsenti H., Harvey-Langton A. (2017). HMGB1/anti-HMGB1 antibodies define a molecular signature of early stages of HIV-Associated Neurocognitive Isorders (HAND). Heliyon.

[15] Marcotte T.D., Deutsch R., Michael B.D., Franklin D., Cookson D.R., Bharti A.R., Grant I., Letendre S.L. (2013). A concise panel of biomarkers identifies neurocognitive functioning changes in HIV-infected individuals. J. Neuroimmune Pharmacol..

[16] Meeker R.B., Poulton W., Markovic-Plese S., Hall C., Robertson K. (2011). Protein changes in CSF of HIV-infected patients: Evidence for loss of neuroprotection. J. Neurovirol..

[17] Schrier R.D., Hong S., Crescini M., Ellis R., Perez-Santiago J., Spina C., Letendre S. (2015). Cerebrospinal fluid (CSF) CD8+ T-cells that express interferon-gamma contribute to HIV associated neurocognitive disorders (HAND). PLoS ONE.

[18] Xing Y., Shepherd N., Lan J., Li W., Rane S., Gupta S.K., Zhang S., Dong J., Yu Q. (2017). MMPs/TIMPs imbalances in the peripheral blood and cerebrospinal fluid are associated with the pathogenesis of HIV-1-associated neurocognitive disorders. Brain Behav. Immun..

[19] Yuan L., Qiao L., Wei F., Yin J., Liu L., Ji Y., Smith D., Li N., Chen D. (2013). Cytokines in CSF correlate with HIV-associated neurocognitive disorders in the post-HAART era in China. J. Neurovirol..

[20] Bilbo S.D., Schwarz J.M. (2009). Early-life programming of later-life brain and behavior: A critical role for the immune system. Front. Behav. Neurosci..

[21] Hsiao E.Y., Patterson P.H. (2012). Placental regulation of maternal-fetal interactions and brain development. Dev. Neurobiol..

[22] Morelli S., Mandal M., Goldsmith L.T., Kashani B.N., Ponzio N.M. (2015). The maternal immune system during pregnancy and its influence on fetal development. Res. Rep. Biol..

[23] White M., Feucht U.D., Duffley E., Molokoane F., Durandt C., Cassol E., Rossouw T., Connor K.L. (2020). Does in utero HIV exposure and the early nutritional environment influence infant development and immune outcomes? Findings from a pilot study in Pretoria, South Africa. Pilot Feasibility Stud..

[24] Hoare J., Myer L., Heany S., Fouche J.P., Phillips N., Zar H.J., Stein D.J. (2020). Cognition, Structural Brain Changes, and Systemic Inflammation in Adolescents Living with HIV on Antiretroviral Therapy. J. Acquir. Immune Defic. Syndr..

[25] Blokhuis C., Peeters C.F.W., Cohen S., Scherpbier H.J., Kuijpers T.W., Reiss P., Kootstra N.A., Teunissen C.E., Pajkrt D. (2019). Systemic and intrathecal immune activation in association with cerebral and cognitive outcomes in paediatric HIV. Sci. Rep..

[26] Benki-Nugent S.F., Martopullo I., Laboso T., Tamasha N., Wamalwa D.C., Tapia K., Langat A., Maleche-Obimbo E., Marra C.M., Bangirana P. (2019). High Plasma Soluble CD163 during Infancy Is a Marker for Neurocognitive Outcomes in Early-Treated HIV-Infected Children. J. Acquir. Immune Defic. Syndr..

[27] Likert R. (1932). A technique for the measurement of attitudes. Arch. Psychol..

[28] Simon A.K., Hollander G.A., McMichael A. (2015). Evolution of the immune system in humans from infancy to old age. Proc. R. Soc. B Biol. Sci..

[29] Fu C., Lu L., Wu H., Shaman J., Cao Y., Fang F., Yang Q., He Q., Yang Z., Wang M. (2016). Placental antibody transfer efficiency and maternal levels: Specific for measles, coxsackievirus A16, enterovirus 71, poliomyelitis I-III and HIV-1 antibodies. Sci. Rep..

[30] Fouda G.G., Martinez D.R., Swamy G.K., Permar S.R. (2018). The Impact of IgG Transplacental Transfer on Early Life Immunity. ImmunoHorizons.

[31] Brenhouse H.C., Schwarz J.M. (2016). Immunoadolescence: Neuroimmune development and adolescent behavior. Neurosci. Biobehav. Rev..

[32] Joachim R.B., Kobzik L. (2018). Why are children more resistant to mortality from severe infections?. Future Microbiol..

[33] Sevenoaks T., Wedderburn C.J., Donald K.A., Barnett W., Zar H.J., Stein D.J., Naudé P.J.W. (2021). Association of maternal and infant inflammation with neurodevelopment in HIV-exposed uninfected children in a South African birth cohort. Brain Behav. Immun..

[34] Kapetanovic S., Giganti M.J., Abzug M.J., Lindsey J.C., Sirois P.A., Montepiedra G., Canniff J., Agwu A., Boivin M.J., Weinberg A. (2021). Plasma biomarker factors associated with neurodevelopmental outcomes in children with perinatal HIV infection and controlled viremia. AIDS.

[35] Kapetanovic S., Griner R., Zeldow B., Nichols S., Leister E., Gelbard H.A., Miller T.L., Hazra R., Mendez A.J., Malee K. (2014). Biomarkers and neurodevelopment in perinatally HIV-infected or exposed youth: A structural equation model analysis. AIDS.

[36] Kapetanovic S., Leister E., Nichols S., Miller T., Tassiopoulos K., Hazra R., Gelbard H.A., Malee K.M., Kammerer B., Mendez A.J. (2010). Relationships between markers of vascular dysfunction and neurodevelopmental outcomes in perinatally HIV-infected youth. AIDS.

[37] Nazmi A., Victora C.G. (2007). Socioeconomic and racial/ethnic differentials of C-reactive protein levels: A systematic review of population-based studies. BMC Public Health.

[38] Hackman D.A., Farah M.J., Meaney M.J. (2010). Socioeconomic status and the brain: Mechanistic insights from human and animal research. Nat. Rev. Neurosci..

[39] Burdo T.H., Weiffenbach A., Woods S.P., Letendre S., Ellis R.J., Williams K.C. (2013). Elevated sCD163 in plasma but not cerebrospinal fluid is a marker of neurocognitive impairment in HIV infection. AIDS.

[40] Williams M.E., Ipser J.C., Stein D.J., Joska J.A., Naudé P.J.W. (2020). Peripheral immune dysregulation in the ART era of HIV-associated neurocognitive impairments: A systematic review. Psychoneuroendocrinology.

[41] Williams M.E., Stein D.J., Joska J.A., Naudé P.J.W. (2021). Cerebrospinal fluid immune markers and HIV-associated neurocognitive impairments: A systematic review. J. Neuroimmunol..

[42] González-Scarano F., Martín-García J. (2005). The neuropathogenesis of AIDS. Nat. Rev. Immunol..

[43] Harezlak J., Buchthal S., Taylor M., Schifitto G., Zhong J., Daar E., Alger J., Singer E., Campbell T., Yiannoutsos C. (2011). Persistence of HIV-associated cognitive impairment, inflammation, and neuronal injury in era of highly active antiretroviral treatment. Aids.

[44] Eckard A.R., Rosebush J.C., O’Riordan M.A., Graves C.C., Alexander A., Grover A.K., Thera Lee S., Habib J.G., Ruff J.H., Chahroudi A. (2017). Neurocognitive dysfunction in HIV-infected youth: Investigating the relationship with immune activation. Antivir. Ther..

[45] Kovacs A. (2009). Early immune activation predicts central nervous system disease in HIV-infected infants: Implications for early treatment. Clin. Infect. Dis..

[46] Kamat A., Lyons J.L., Misra V., Uno H., Morgello S., Singer E.J., Gabuzda D. (2012). Monocyte activation markers in cerebrospinal fluid associated with impaired neurocognitive testing in advanced HIV infection. J. Acquir. Immune Defic. Syndr..

[47] Rubin L.H., Benning L., Keating S.M., Norris P.J., Burke-Miller J., Savarese A., Kumanan K.N., Awadalla S., Springer G., Anastos K. (2018). Variability in C-reactive protein is associated with cognitive impairment in women living with and without HIV: A longitudinal study. J. Neurovirol..

[48] Wilmshurst J.M., Hammond C.K., Donald K., Hoare J., Cohen K., Eley B. (2018). NeuroAIDS in children. Handbook of Clinical Neurology.

[49] Blokhuis C., Kootstra N.A., Caan M.W.A., Pajkrt D. (2016). Neurodevelopmental delay in pediatric HIV/AIDS: Current perspectives. Neurobehav. HIV Med..

[50] Sharer L.R., Cho E.S. (1989). Neuropathology of HIV infection: Adults versus children. Prog. AIDS Pathol..

[51] Tardieu M., Le Chenadec J., Persoz A., Meyer L., Blanche S., Mayaux M.J. (2000). HIV-1-related encephalopathy in infants compared with children and adults. Neurology.

[52] Khiati A., Chaloin O., Muller S., Tardieu M., Horellou P. (2010). Induction of monocyte chemoattractant protein-1 (MCP-1/CCL2) gene expression by human immunodeficiency virus-1 Tat in human astrocytes is CDK9 dependent. J. Neurovirol..

[53] Mitchell W. (2001). Neurological and developmental effects of HIV and AIDS in children and adolescents. Ment. Retard. Dev. Disabil. Res. Rev..

[54] Van Rie A., Harrington P.R., Dow A., Robertson K. (2007). Neurologic and neurodevelopmental manifestations of pediatric HIV/AIDS: A global perspective. Eur. J. Paediatr. Neurol..

[55] Tobin N.H., Aldrovandi G.M. (2013). Immunology Of Pediatric HIV Infection. Immunol. Rev..

[56] Clifford D.B., Ances B.M. (2013). HIV-associated neurocognitive disorder. Lancet Infect. Dis..

[57] Hattab S., Guiguet M., Carcelain G., Fourati S., Guihot A., Autran B., Caby F., Marcelin A.G., Costagliola D., Katlama C. (2015). Soluble biomarkers of immune activation and inflammation in HIV infection: Impact of 2 years of effective first-line combination antiretroviral therapy. HIV Med..

[58] Oliveira M.F., Chaillon A., Nakazawa M., Vargas M., Letendre S.L., Strain M.C., Ellis R.J., Morris S., Little S.J., Smith D.M. (2017). Early Antiretroviral Therapy Is Associated with Lower HIV DNA Molecular Diversity and Lower Inflammation in Cerebrospinal Fluid but Does Not Prevent the Establishment of Compartmentalized HIV DNA Populations. PLoS Pathog..

[59] Rajasuriar R., Wright E., Lewin S.R. (2015). Impact of antiretroviral therapy (ART) timing on chronic immune activation/inflammation and end-organ damage. Curr. Opin. HIV AIDS.

[60] Boivin M.J., Ruel T.D., Boal H.E., Bangirana P., Cao H., Eller L.A., Charlebois E., Havlir D.V., Kamya M.R., Achan J. (2010). HIV-subtype A is associated with poorer neuropsychological performance compared with subtype D in antiretroviral therapy-naive Ugandan children. AIDS.

[61] Rao V.R., Neogi U., Talboom J.S., Padilla L., Rahman M., Fritz-French C., Gonzalez-Ramirez S., Verma A., Wood C., Ruprecht R.M. (2013). Clade C HIV-1 isolates circulating in Southern Africa exhibit a greater frequency of dicysteine motif-containing Tat variants than those in Southeast Asia and cause increased neurovirulence. Retrovirology.

[62] Gandhi N., Saiyed Z., Thangavel S., Rodriguez J., Rao K.V.K., Nair M.P.N. (2009). Differential effects of HIV type 1 clade B and clade C Tat protein on expression of proinflammatory and antiinflammatory cytokines by primary monocytes. AIDS Res. Hum. Retrovir..

[63] Semrud-Clikeman M., Romero R.A.A., Prado E.L., Shapiro E.G., Bangirana P., John C.C. (2017). Selecting measures for the neurodevelopmental assessment of children in low- and middle-income countries. Child Neuropsychol..

[64] Çetin I., Çetin A., Şen A., Cimen L., Çimen B., Savas G., Oztürk A., Koker M.Y. (2018). Comparison of ELISA and flow cytometry for measurement of interleukin-1 beta, interleukin-6 and tumor necrosis factor-α. Turk. J. Biochem..

[65] Ungaro C.T., Wolfe A.S., Brown S.D. (2020). Comparison of serum cytokine measurement techniques between ELISA vs Multiplex. FASEB J..

[66] Lasseter H.C., Provost A.C., Chaby L.E., Daskalakis N.P., Haas M., Jeromin A. (2020). Cross-platform comparison of highly sensitive immunoassay technologies for cytokine markers: Platform performance in post-traumatic stress disorder and Parkinson’s disease. Cytokine X.

