# Supplementary material for: Immune Dysregulation Is Associated with Neurodevelopment and Neurocognitive Performance in HIV Pediatric Populations—A Scoping Review
Source: Viruses. 2021 Dec 18;13(12):2543. doi: 10.3390/v13122543 (PMC8706807; doi:10.3390/v13122543)
Supplement: Supplementary file 1 [file viruses-13-02543-s001.zip › viruses-1425853-supplementary.pdf]

Table S1. (1) Quality assessment of studies by MEW.

Reference	Question 1	Question 2			Rating
			Regimen	Duration of treatment	
[26]*	2	1	✓	✓	Intermediate
[25]	2	1	✓	✓	Intermediate
[24]	2	1	✓	✓	Intermediate
[31]	1	1	✓		Intermediate
[30]	1	1	✓		Intermediate
[29]*	2	1	✓	✓	Intermediate
[28]*	2	2	✓	✓	High

Table S1. (2) Quality assessment of studies by AJVR.

Reference	Question 1	Question 2			Rating
			Regimen	Duration of treatment	
[26]*	**2**	1	✓	✓	Intermediate
[25]	1	1	✓	✓	Intermediate
[24]	1	1	✓	✓	Intermediate
[31]	1	1	✓		Intermediate
[30]	2	1	✓		Intermediate
[29]*	2	1	✓	✓	Intermediate
[28]*	2	2	✓	✓	High

*=Longitudinal studies. Questions 1-3 were classified as follows: 1) Did the study report on potential confounders and were these controlled for upon statistical analysis, including the controlling of multiple comparisons? 2) Did the study report on the background information of the mother and child which may have affected inflammatory levels and neurocognitive performance/development in the pediatric population (*i.e.*, maternal substance abuse, prematurity, ART duration and regimens and low birth weight). Each question was rated for 0 = no, 1 = partly and 2 = yes. Studies that addressed all the above questions and had a total rating of 4 were classified as high quality. Studies with a rating between 1 and 3 were considered as intermediate-quality and less than 1 as low quality.

Table S2. Studies reporting a relationship of immune markers and HIV-associated neurocognitive performance/development in pediatric populations when stratified according to viral suppression.

Reference	Virally suppressed	Non-virally suppressed	Association with neurocognitive impairment/development	
			Yes	No
[26]*		✓	✓	
[25]		✓	✓	
[24]	✓		✓	
[31]		✓		✓
[30]		✓	✓	
[29]*	✓		✓	

*longitudinal studies.

Table S3. Studies reporting a relationship with HIV-associated neurocognitive performance when stratified according to nadir/current CD4 count.

Reference	<200 cells/µL		>200 cells/µL		Association with neurocognitive impairment/development	
	Nadir	Current	Nadir	Current	Yes	No
[24]				✓	✓	
[31]			✓			✓
[30]			✓	✓	✓	
